# BRICHOS - a superfamily of multidomain proteins with diverse functions

**DOI:** 10.1186/1756-0500-2-180

**Published:** 2009-09-11

**Authors:** Joel Hedlund, Jan Johansson, Bengt Persson

**Affiliations:** 1 IFM Bioinformatics, Linköping University, S-581 83 Linköping, Sweden; 2 SLU, Dept of Anatomy, Physiology and Biochemistry, The Biomedical Centre, Box 575, S-751 23 Uppsala, Sweden; 3 Department of Cell and Molecular Biology (CMB), Karolinska Institutet, S-171 77 Stockholm, Sweden

## Abstract

**Background:**

The BRICHOS domain has been found in 8 protein families with a wide range of functions and a variety of disease associations, such as respiratory distress syndrome, dementia and cancer. The domain itself is thought to have a chaperone function, and indeed three of the families are associated with amyloid formation, but its structure and many of its functional properties are still unknown.

**Findings:**

The proteins in the BRICHOS superfamily have four regions with distinct properties. We have analysed the BRICHOS proteins focusing on sequence conservation, amino acid residue properties, native disorder and secondary structure predictions. Residue conservation shows large variations between the regions, and the spread of residue conservation between different families can vary greatly within the regions. The secondary structure predictions for the BRICHOS proteins show remarkable coherence even where sequence conservation is low, and there seems to be little native disorder.

**Conclusions:**

The greatly variant rates of conservation indicates different functional constraints among the regions and among the families. We present three previously unknown BRICHOS families; group A, which may be ancestral to the ITM2 families; group B, which is a close relative to the gastrokine families, and group C, which appears to be a truly novel, disjoint BRICHOS family. The C-terminal region of group C has nearly identical sequences in all species ranging from fish to man and is seemingly unique to this family, indicating critical functional or structural properties.

## Findings

The BRICHOS domain has been found in proteins with a wide range of functions and disease associations [1]. There are 8 known families; the cancer associated GKN1, GKN2 and LECT1, the three dementia associated ITM2 families, the respiratory disease associated proSP-C, and TNMD. There is little sequence identity between the families, the proteins are generally cleaved to produce their active forms, and there are no structures even for remote homologues in the PDB database.

**Figure 1:**
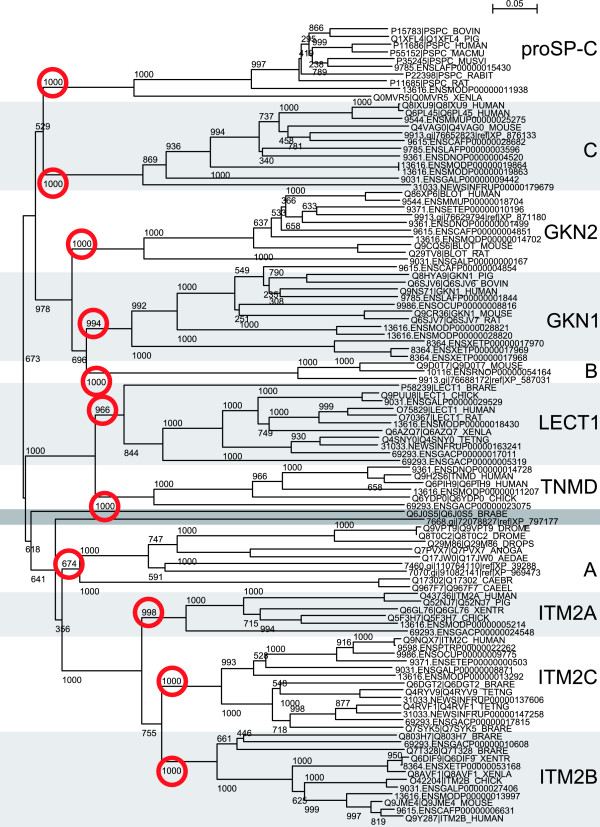
**Dendrogram of the BRICHOS superfamily**. 12 groups are clearly distinguished; proSP-C (pulmonary surfactant protein C precursor), group C, GKN2 and GKN1 (gastrokine-2 and -1), group B, LECT1 (chondromodulin-1), TNMD (tenomodulin), the divergent group, group A, and ITM2A, ITM2C and ITM2B (integral membrane protein 2 A, C and B). UniProtKB sequences are denoted by accession number and identifier, e.g: O43736|ITM2A_HUMAN. GenomeLKPG sequences are denoted by their external identifier (Ensembl or NCBI) prepended with the organism's NCBI Taxonomic identifier, e.g. 13618.ENSMODP00000005214. Red circles highlight the bootstrap numbers for each family. Only sequences with less than 90% sequence identities are shown.

Searching UniProtKB [2] and GenomeLKPG (translated public domain genomes, personal communication with Anders Bresell, Linköping University) revealed 309 BRICHOS proteins. These clearly separate into 12 groups; the 8 previously known families, 3 novel families, and one divergent group of only two sequences (cf Fig. 1).

Group A is a novel family that clusters closely with the ITM2 families, albeit with low bootstrap values. The position in the dendrogram indicates that group A with its primarily insect and *Caenorhabditis *sequences may be ancestral to the ITM2 families.

The divergent group branches off before group A, and its echinoderm and amphioxus sequences are compatible with an ancestral nature.

GKN1, GKN2 and group B are closely related families that are also colocalised in the genome, suggesting that group B may be a third type of gastrokine. Group B is found only in mouse, rat, cow and dolphin, while GKN1 and GKN2 are found in a wide range of mammals (also frog and chicken, respectively).

LECT1 and TNMD are widespread in vertebrates, from fish through armadillo and elephant to human, though TNMD has so far not been reported in frog.

Group C is another novel family. Neither this nor proSP-C clusters strongly with any other family, but both are present in tetrapods. While group C is found in fish but not frog, the opposite is true for proSP-C which is consistent with its role as a pulmonary surfactant constituent.

BRICHOS proteins have four regions; hydrophobic, linker, BRICHOS and C-terminal (length distributions shown in Table 1). The hydrophobic region is most often a transmembrane segment (predictions and [3]) but may be a signal peptide in GKN1 and GKN2 [4]. In proSP-C it functions as both [5].

**Table 1:** Length distributions for different regions of BRICHOS proteins

	**Length**			
	
**Region**	**min**	**max**	**median**	**stddev**
Hydrophobic	12	33	26	4.7
Linker	24	105	42	14.5
BRICHOS	83	104	93	2.5
C-terminal	29	149	38	35.2

Numbers give minima, maxima, medians and standard deviations for the region lengths. The C-terminal region is absent from the proSP-C family, and consequently the length characteristics for this region are shown excluding proSP-C.

All families except GKN1 and GKN2 have an additional N-terminal region that is poorly conserved, highly variable in length and likely separated from the other regions by a membrane. This region is not further investigated in this study.

All statements regarding the C-terminal region exclude proSP-C since it is absent from this family.

### Conservation and secondary structure

**Figure 2:**
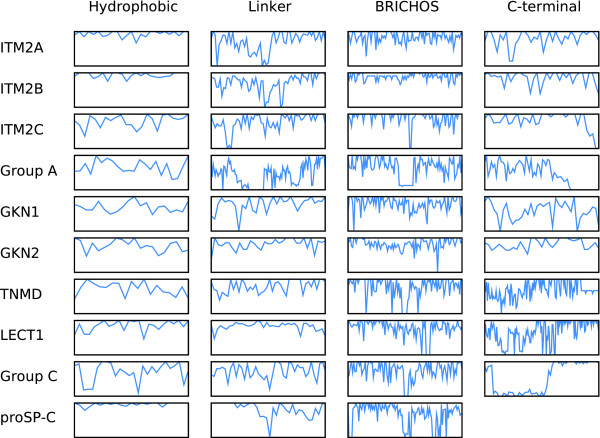
**Conservation profiles of BRICHOS proteins**. Each row describes one BRICHOS family and each column describes one region. The vertical axis in each plot shows *cscores *from 0% to 100%, and the horizontal axes span the length of the corresponding family and region.

As shown in Table 2, 3, 4 and 5, residue conservation differs considerably among the regions. The spread in *ID* (average pairwise percent identities) for the hydrophobic region is wide, from 26% in group A to 96% in proSP-C, indicating drastically different functional constraints. Conversely for the BRICHOS region, all families have 51-83% *ID*, indicating similar functions among the families. The remaining regions show wide *ID *spreads. The *GC *values (group conservation, Table 2, 3, 4 and 5) show the largest spread for the hydrophobic region, with highest values for proSP-C and ITM2A. The linker region shows the lowest *GC *values (8-46%). Despite high numbers for *cscore *and *ID*, the LECT1 linker region shows an extremely low *GC *value (8%) compared to its other regions (37-48%). The three ITM2 families show similar values in all regions except the hydrophobic one, whose 36-86% *GC *might indicate differering structural constraints. The regional conservation differ considerably between families (cf Fig. 2). proSP-C has its highest *cscore *in the hydrophobic region (96%) while for group C it is highest in the C-terminal region (76%). The hydrophobic region is the most conserved in ITM2A while it is the least conserved in group C.

**Table 2:** Conservation measures in the hydrophobic region

**Family**	***n***	***cscore***	***ID***	***GC***
ITM2A	8	92	82	86
ITM2B	13	93	80	64
ITM2C	16	79	50	36
Group A	9	66	26	28
GKN1	11	69	38	25
GKN2	8	77	42	26
TNMD	5	72	44	41
LECT1	13	84	74	49
group C	11	70	50	17
proSP-C	12	96	96	91

Conservation the hydrophobic region for the different BRICHOS families, shown in percent. *cscore *denotes average conservation score. *ID *denotes median pairwise sequence identity. *GC *denotes the proportion of positions conserved either strictly or within the groups of highly similar residues {DE}, {KR}, {FILMV} or {ST}. *n *denotes the number of sequences present in the underlying set. *GC *is a stricter measure of functional conservation, but may be more sensitive to atypical sequences.

**Table 3:** Conservation measures in the linker region

**Family**	***cscore***	***ID***	***GC***
ITM2A	70	58	42
ITM2B	71	53	30
ITM2C	77	56	36
Group A	42	23	26
GKN1	78	57	30
GKN2	81	62	29
TNMD	79	71	37
LECT1	82	63	8
group C	72	54	20
proSP-C	82	78	46

Conservation the linker region for the different BRICHOS families, shown in percent. Column headings as explained in Table 2.

**Table 4:** Conservation measures in the BRICHOS region

**Family**	***cscore***	***ID***	***GC***
ITM2A	83	67	58
ITM2B	89	83	71
ITM2C	89	82	71
Group A	66	57	39
GKN1	79	53	35
GKN2	82	74	50
TNMD	77	70	55
LECT1	78	64	37
group C	75	51	29
proSP-C	67	67	30

Conservation the BRICHOS region for the different BRICHOS families, shown in percent. Column headings as explained in Table 2.

**Table 5:** Conservation measures in the C-terminal region

**Family**	***cscore***	***ID***	***GC***
ITM2A	79	55	52
ITM2B	85	71	62
ITM2C	81	67	51
Group A	45	30	26
GKN1	58	26	16
GKN2	81	60	23
TNMD	69	32	32
LECT1	67	48	42
group C	94	87	76

Conservation the C-terminal region for the different BRICHOS families, shown in percent. Column headings as explained in Table 2. The numbers for group C are presented excluding the insertions shown in Fig. 5.

**Figure 3:**
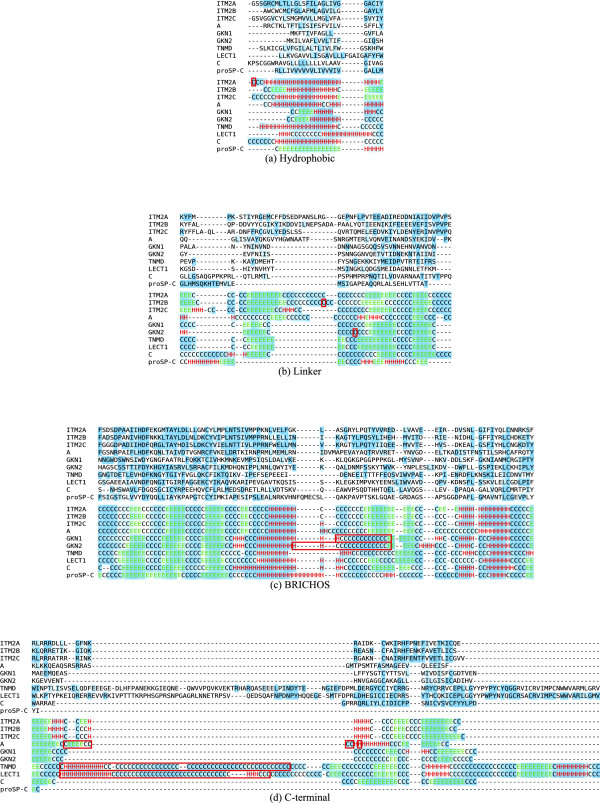
**Conservation, secondary structure and native disorder**. The upper half of each figure shows *GC *positions within each family in blue (strictly conserved in dark blue). The lower half shows secondary structure predictions for the representative sequences in colored letters (red H for helix, green E for strand, black C for coil) while the background shading indicates prediction reliability (the stronger the better). Red rectangles indicate native disorder. The alignment is an excerpt from a full alignment of the superfamily, showing only one human representative from each family, and a *Caenorhabditis *sequence for group A, suppressing any resulting fully gapped positions.

Fig. 3 shows alignments for each region. Remarkably, although the degree of conservation is high in individual families, only three residues are completely conserved in the superfamily; D144, C160 and C219 (human ITM2A numbering), all in the BRICHOS region. The corresponding cysteines in proSP-C form an internal disulphide bridge [6] which could be the case for all families. C244 and C261 in the C-terminal region are strictly conserved in all families, except in group A where they are absent from all sequences, and in TNMD where one stickleback sequence has tyrosine replacing the latter cysteine. However since the stickleback genome project is still ongoing, this might represent a sequencing error. Thus, these cysteines might also form a disulphide bridge.

The structure is still unknown for the BRICHOS proteins. However while the degree of conservation across the superfamily is low there is remarkable coherence in secondary structure, not only in the BRICHOS domain. Also, the few natively disordered regions are with few exceptions found N-terminally of the hydrophobic region, indicating that the proteins may have otherwise well defined tertiary structures.

#### Hydrophobic region

The hydrophobic region is strongly predicted to be helical (Fig. 3a). Notable exceptions are GKN1 and GKN2 where the first 6 residues of the predicted signal peptide show strand tendencies. The proSP-C prediction surprisingly shows strand tendencies, disagreeing with experimental evidence of a helical structure [7].

The remarkably high conservation in ITM2A, ITM2B and proSP-C (Fig. 2), and the high number of strictly conserved valines in proSP-C, are unusual for a transmembrane segment, indicating possible additional roles (e.g. protein interactions). The high degree of conservation in proSP-C is expected since it corresponds to mature SP-C [5,8]. No interactions with other proteins have been described for mature helical SP-C, except for possible homodimerisation [9].

#### Linker region

The linker region (Fig. 3b) favours coil and strand conformations and shows a lower degree of conservation, except in proSP-C where the high degree of conservation in the hydrophobic region extends into this region.

#### BRICHOS region

The BRICHOS region shows the highest degree of conservation near the strictly conserved aspartic acid and first cysteine residues, but is less conserved in the C-terminal half (Fig. 3c). The initial section is predicted to form three short strands interspersed with short coils. The remainder is dominated by two helices that are conserved in all families, separated by a coil-strand-coil region. Surprisingly, proSP-C instead shows slight helical tendencies here.

**Figure 4:**
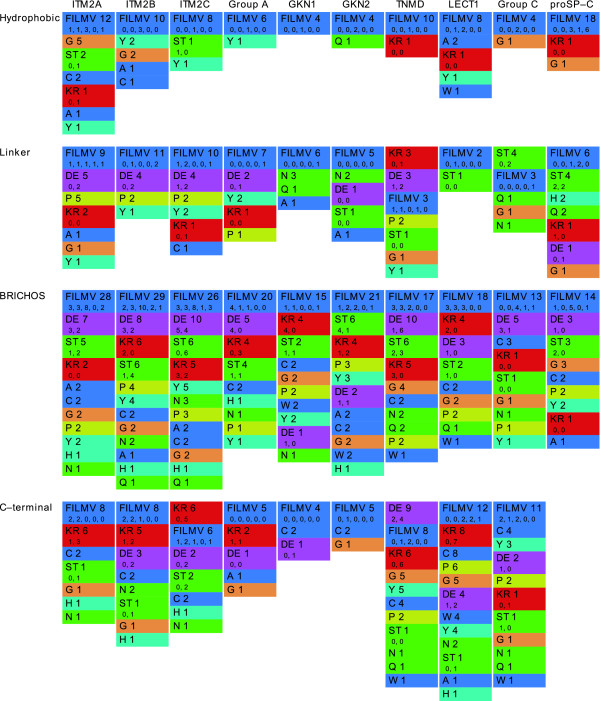
**Ranked residue conservation**. Conserved residues and groups of residues in BRICHOS families by region, ordered by descending number of observations. The observations for *GC *groups are aggregated, showing the number of strictly conserved residues under the totals, in the corresponding order.

The BRICHOS domain of ITM2 has a conserved net negative charge correlated with a conserved net positive charge in the C-terminal region, being most extreme for ITM2A with net charges -5 and +6 in the different regions (Fig. 4). This characteristic is shared by group A, but less pronounced. Furthermore, group A lacks the remarkably high number of conserved hydrophobic residues in the ITM2 BRICHOS domains. It is more similar to the other families in this respect, in accordance with group A being ancestral to ITM2.

LECT1 and TNMD are similar in many aspects but have drastically different conserved net charges, especially in the BRICHOS domain and C-terminal region.

GKN1, GKN2 and group B may have a central natively disordered segment coinciding with a strongly predicted coiled segment (cf Fig. 3c, group B not shown). This is surprising since this characteristic is not shared by the other families.

#### C-terminal region

**Figure 5:**
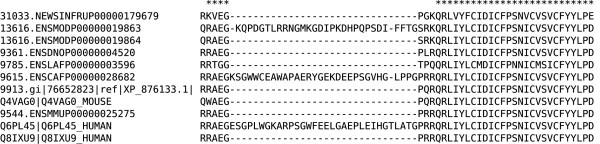
**Multiple sequence alignment of the C-terminal region of group C**. Asterisks denote positions with at most one divergent residue. Sequence labels follow the same format as in Fig. 1.

The C-terminal region is extremely well conserved in group C (Fig. 5) with nearly identical sequences in all species ranging from fish to man. However, three sequences have a poorly conserved insertion of 30-odd residues whose boundaries correlate with splice sites for surrounding exons, potentially stemming from spliceoforms or incorrect exon predictions. Excluding these increases the average *cscore *to from 52% to 94%.

GKN1 and GKN2 show a low degree of conservation in this region, as does group A, which is surprising given its similarity to the well conserved ITM2 families.

The C-terminal region is well conserved in ITM2, TNMD and LECT1, although LECT1 and TNMD have a long and less conserved insertion (Fig. 3d). These insertions may be largely natively disordered, however while most of these segments are likely coiled, the initial parts of the segments are ascribed a moderate probability of being helical. Group A also shows signs of native disorder in this segment, contrarily to ITM2.

Transmembrane predictors ascribe a moderate probability for group C to have a transmembrane helix here, which would be unexpected considering its predicted strand structure and extreme conservation.

Surprisingly, conservation in LECT1, TNMD and group C increases near the C-terminus (Fig. 2). The decrease for TNMD stems from a truncated stickleback sequence. This part contains four strictly conserved cysteines which could potentially form disulphide bridges or coordinate metal ions.

The C-terminal regions of the BRICHOS proteins have no detectable homologues in UniProtKB, making the well conserved C-terminal regions of group C, LECT1 and TNMD unique to this superfamily and especially interesting for further studies.

### Disease-related mutations

Several mutations in the proSP-C BRICHOS region correlate with lung disease. Notably, N138T and N186S increase susceptibility to perinatal RDS [10] while substituting asparagine for the residue type that is most frequent in orthologues. Three substitutions are associated with SMDP2. A116D affects a strictly conserved position (except one arginine in frog). R167Q is a naturally occurring polymorphism and affects a non-conserved position. L188Q affects a strictly conserved position and is found in association with familial interstitial lung disease [11]. Also, mutant proSP-C L188Q does not function as a chaperone for unfolded SP-C [8].

The linker regions also has disease related substitutions. E66L is associated with abnormal targeting to early endosomes and likely toxic gain of function [12], and affects a strictly conserved position. I73T causes abnormal trafficking and accumulation of aberrantly processed proSPC within alveoli [12]. Orthologues hold isoleucine, methionine and leucine, however positions 71-72 are strictly conserved, suggesting importance of this segment. Notably, protein sorting predictions [13-16] are unchanged following the substitution, and thus disagree with experimental results.

In ITM2B, two stop codon disruptions associated with dementia yield amyloidogenic proteins elongated by 11 residues; duplication of 10 nucleotides between the penultimate and final translated codons in FDD [17], and a single base substitution in FBD [18].

In the BRICHOS region of GKN1, E104T is associated with breast cancer [19] and is conserved to lysine in all other species (except asparagine in cow, and glutamine in mouse and rat).

## Methods

Sequences were collected using HMMER [20], both with the BRICHOS model from PfamA [21] and a custom HMMER model with equal specificity and slightly higher sensitivity. Partial sequences were manually removed. MSAs were made using dialign-t [22] and mafft L-INS-i [23]. Neighbour joining dendrograms were built using ClustalX [24]. Transmembrane topology was predicted using Phobius [25] and TMHMM [26]. Secondary structure elements were predicted using Prof [27], PredictProtein [28] and Psipred [29]. DISOPRED2 was used for native disorder prediction [30]. Due to its small size, group B was excluded from quantitative conservation comparisons.

### Conservation scoring

The *cscore *is similar to the ClustalX qscore (see source code), being a diminishing function of the average euclidean distance to the centroid for the substitution score vectors for the symbols in the MSA. However, this algorithm uses a linear distance-to-score transform and penalises partially gapped positions less severely than does the ClustalX variant.

In the *cscore *algorithm, the centroid *C*_*i *_is calculated using the expression

(1)

*N *denotes the number of sequences, *M*_*i, j *_the symbol in sequence *j *at position *i*, *S*_*x *_the score vector for residue type *x*, *σ *the set of *n *symbols described by *S*, and *N*_*u *_the number of symbols in the position that are not described by *S*. Thus, unlike ClustalX, gaps and other symbols not in *σ *do not contribute to the placement of the centroid. Rather, when calculating the average euclidean distance *d*_*i *_to the centroid, these symbols are assigned the penalty distance

(2)

where *d*_*λ *_is half the maximum distance between any two vectors in *S*. The transform from distance to cscore *c*_*i *_is not exponential as in ClustalX, but rather a partially linear function of *d*_*i*_

(3)

*d*_*u *_is defined so that *c*_*i *_= 0 for positions where only one residue is in *σ*. Consequently, *d*_*i *_can be greater than *d*_*λ *_in exceptional cases (e.g. fully gapped positions), and the nonlinearity in equation 3 will assign *c*_*i *_= 0 to such positions.

## Conclusions

We have characterised the BRICHOS superfamily and its four regions with distinct properties. We find large variation in conservation in both regions and families, which implies differences in functional constraints. Secondary structure elements are seemingly well conserved even in regions with low residue conservation. This coupled with the apparent low degree of predicted native disorder indicates that tertiary structure may be similarly conserved.

We show that most of the known disease related mutations are in highly conserved positions, and that in two cases related to proSP-C and RDS, it is the substitution from the atypical human asparagines to the otherwise strictly conserved threonine and serine that are associated with disease.

We have identified three novel BRICHOS families; group A, which may be ancestral to the ITM2 families; group B, which is a close relative to the GKN families, and group C, which appears to be a truly novel, disjoint BRICHOS family. The C-terminal region of group C is unique to this family, with nearly identical sequences in all species ranging from fish to man, indicating critical functional or structural properties.

## Abbreviations

**BRICHOS families**: GKN: Gastrokine, two families (GKN1 and GKN2); ITM: Integral transmembrane protein, three families (ITM2A, ITM2B and ITM2C); LECT1: Chondromodulin-1 precursor; proSP-C: Pulmonary surfactant protein C precursor; TNMD: Tenomodulin-1. **Other**: FBD: Familial British dementia; FDD: Familial Danish dementia; GC: Group conservation, proportion of positions conserved strictly or within groups of highly similar residues; ID: Average percent pairwise sequence identities; MSA: Multiple sequence alignment; RDS: Respiratory distress syndrome; SMDP2: Surfactant metabolism dysfunction, pulmonary.

## Competing interests

The authors declare that they have no competing interests.

## Authors' contributions

JH performed HMM creation and database searches, performed the sequence analyses, created the *cscore *conservation scoring algorithm and drafted the manuscript. JJ initiated the study and helped to draft the manuscript. BP supervised the study, participated in its design and coordination and helped to draft the manuscript. All authors have read and approved the final manuscript.

## References

[B1] Sanchez-Pulido L, Devos D, Valencia A (2002). BRICHOS: a conserved domain in proteins associated with dementia, respiratory distress and cancer. Trends Biochem Sci.

[B2] The Uniprot consortium (2007). The Universal Protein Resource (UniProt). Nucleic Acids Res.

[B3] Martin L, Fluhrer R, Reiss K, Kremmer E, Saftig P, Haass C (2008). Regulated intramembrane proteolysis of Bri2 (Itm2b) by ADAM10 and SPPL2a/SPPL2b. J Biol Chem.

[B4] Bairoch A, Boeckmann B, Ferro S, Gasteiger E (2004). Swiss-Prot: juggling between evolution and stability. Brief Bioinform.

[B5] Keller A, Eistetter HR, Voss T, Schafer KP (1991). The pulmonary surfactant protein C (SP-C) precursor is a type II transmembrane protein. Biochem J.

[B6] Casals C, Johansson H, Saenz A, Gustafsson M, Alfonso C, Nordling K, Johansson J (2008). C-terminal, endoplasmic reticulum-lumenal domain of prosurfactant protein C - structural features and membrane interactions. FEBS J.

[B7] Kallberg Y, Gustafsson M, Persson B, Thyberg J, Johansson J (2001). Prediction of amyloid fibril-forming proteins. J Biol Chem.

[B8] Johansson H, Nordling K, Weaver TE, Johansson J (2006). The Brichos domain-containing C-terminal part of pro-surfactant protein C binds to an unfolded poly-val transmembrane segment. J Biol Chem.

[B9] Luy B, Diener A, Hummel RP, Sturm E, Ulrich WR, Griesinger C (2004). Structure and potential C-terminal dimerization of a recombinant mutant of surfactant-associated protein C in chloroform/methanol. Eur J Biochem.

[B10] Lahti M, Marttila R, Hallman M (2004). Surfactant protein C gene variation in the Finnish population-association with perinatal respiratory disease. Eur J Hum Genet.

[B11] Thomas AQ, Lane K, Phillips J, Prince M, Markin C, Speer M, Schwartz DA, Gaddipati R, Marney A, Johnson J, Roberts R, Haines J, Stahlman M, Loyd JE (2002). Heterozygosity for a surfactant protein C gene mutation associated with usual interstitial pneumonitis and cellular nonspecific interstitial pneumonitis in one kindred. Am J Respir Crit Care Med.

[B12] Stevens PA, Pettenazzo A, Brasch F, Mulugeta S, Baritussio A, Ochs M, Morrison L, Russo SJ, Beers MF (2005). Nonspecific interstitial pneumonia, alveolar proteinosis, and abnormal proprotein trafficking resulting from a spontaneous mutation in the surfactant protein C gene. Pediatr Res.

[B13] Nakai K, Kanehisa M (1992). A knowledge base for predicting protein localization sites in eukaryotic cells. Genomics.

[B14] Lu Z, Szafron D, Greiner R, Lu P, Wishart DS, Poulin B, Anvik J, Macdonell C, Eisner R (2004). Predicting subcellular localization of proteins using machine-learned classifiers. Bioinformatics.

[B15] Jin YH, Niu B, Feng KY, Lu WC, Cai YD, Li GZ (2008). Predicting subcellular localization with AdaBoost Learner. Protein Pept Lett.

[B16] Emanuelsson O, Brunak S, von Heijne G, Nielsen H (2007). Locating proteins in the cell using TargetP, SignalP and related tools. Nat Protoc.

[B17] Vidal R, Revesz T, Rostagno A, Kim E, Holton JL, Bek T, Bojsen-Moller M, Braendgaard H, Plant G, Ghiso J, Frangione B (2000). A decamer duplication in the 3' region of the BRI gene originates an amyloid peptide that is associated with dementia in a Danish kindred. Proc Natl Acad Sci USA.

[B18] Vidal R, Frangione B, Rostagno A, Mead S, Revesz T, Plant G, Ghiso J (1999). A stop-codon mutation in the BRI gene associated with familial British dementia. Nature.

[B19] Sjoblom T, Jones S, Wood LD, Parsons DW, Lin J, Barber TD, Mandelker D, Leary RJ, Ptak J, Silliman N, Szabo S, Buckhaults P, Farrell C, Meeh P, Markowitz SD, Willis J, Dawson D, Willson JK, Gazdar AF, Hartigan J, Wu L, Liu C, Parmigiani G, Park BH, Bachman KE, Papadopoulos N, Vogelstein B, Kinzler KW, Velculescu VE (2006). The consensus coding sequences of human breast and colorectal cancers. Science.

[B20] Eddy SR (1998). Profile hidden Markov models. Bioinformatics.

[B21] Finn RD, Mistry J, Schuster-Bockler B, Griffiths-Jones S, Hollich V, Lassmann T, Moxon S, Marshall M, Khanna A, Durbin R, Eddy SR, Sonnhammer EL, Bateman A (2006). Pfam: clans, web tools and services. Nucleic Acids Res.

[B22] Subramanian AR, Weyer-Menkhoff J, Kaufmann M, Morgenstern B (2005). DIALIGN-T: an improved algorithm for segment-based multiple sequence alignment. BMC Bioinformatics.

[B23] Katoh K, Kuma K, Toh H, Miyata T (2005). MAFFT version 5: improvement in accuracy of multiple sequence alignment. Nucleic Acids Res.

[B24] Thompson JD, Gibson TJ, Plewniak F, Jeanmougin F, Higgins DG (1997). The CLUSTAL_X windows interface: flexible strategies for multiple sequence alignment aided by quality analysis tools. Nucleic Acids Res.

[B25] Kall L, Krogh A, Sonnhammer EL (2004). A combined transmembrane topology and signal peptide prediction method. J Mol Biol.

[B26] Moller S, Croning MD, Apweiler R (2001). Evaluation of methods for the prediction of membrane spanning regions. Bioinformatics.

[B27] Ouali M, King RD (2000). Cascaded multiple classifiers for secondary structure prediction. Protein Sci.

[B28] Rost B, Yachdav G, Liu J (2004). The PredictProtein server. Nucleic Acids Res.

[B29] Bryson K, McGuffin LJ, Marsden RL, Ward JJ, Sodhi JS, Jones DT (2005). Protein structure prediction servers at University College London. Nucleic Acids Res.

[B30] Ward JJ, Sodhi JS, McGuffin LJ, Buxton BF, Jones DT (2004). Prediction and functional analysis of native disorder in proteins from the three kingdoms of life. J Mol Biol.

